# Supplementary material for: Resolving the full spectrum of human genome variation using Linked-Reads
Source: Genome Res. 2019 Apr;29(4):635–45. doi: 10.1101/gr.234443.118 (PMC6442396; doi:10.1101/gr.234443.118)
Supplement: Supplemental Material [file supp_gr.234443.118_Supplemental_longranger-2.2.zip › longranger-2.2.tar.bz2!/lr2.2-public/lib/stan/cmdstan/stan/doc/api/html/classstan_1_1mcmc_1_1base__leapfrog.html]

Stan: stan::mcmc::base\_leapfrog< H, P > Class Template Reference


|  |  |
| --- | --- |
|  | Stan  2.5.0  probability, sampling & optimization |


- Main Page
- Related Pages
- Namespaces
- Classes
- Files

- Class List
- Class Index
- Class Hierarchy
- Class Members

All Classes Namespaces Files Functions Variables Typedefs Enumerations Enumerator Friends Macros Pages

- stan
- mcmc
- base\_leapfrog

Public Member Functions |
List of all members

stan::mcmc::base\_leapfrog< H, P > Class Template Referenceabstract

`#include <base_leapfrog.hpp>`

Inheritance diagram for stan::mcmc::base\_leapfrog< H, P >:

|  |  |
| --- | --- |
| Public Member Functions | |
|  | base\_leapfrog (std::ostream \*o) |
|  | |
| void | evolve (P &z, H &hamiltonian, const double epsilon) |
|  | |
| void | verbose\_evolve (P &z, H &hamiltonian, const double epsilon) |
|  | |
| virtual void | begin\_update\_p (P &z, H &hamiltonian, double epsilon)=0 |
|  | |
| virtual void | update\_q (P &z, H &hamiltonian, double epsilon)=0 |
|  | |
| virtual void | end\_update\_p (P &z, H &hamiltonian, double epsilon)=0 |
|  | |
| Public Member Functions inherited from stan::mcmc::base\_integrator< H, P > | |
|  | base\_integrator (std::ostream \*o) |
|  | |

|  |  |
| --- | --- |
| Additional Inherited Members | |
| Protected Attributes inherited from stan::mcmc::base\_integrator< H, P > | |
| std::ostream \* | out\_stream\_ |
|  | |

## Detailed Description

### template<typename H, typename P> class stan::mcmc::base\_leapfrog< H, P >

Definition at line 13 of file base\_leapfrog.hpp.

## Constructor & Destructor Documentation

template<typename H, typename P>

|  |  |  |  |  |  |
| --- | --- | --- | --- | --- | --- |
| stan::mcmc::base\_leapfrog< H, P >::base\_leapfrog | ( | std::ostream \* | *o* | ) |  |

inline

Definition at line 17 of file base\_leapfrog.hpp.

## Member Function Documentation

template<typename H, typename P>

|  |  |  |  |
| --- | --- | --- | --- |
| virtual void stan::mcmc::base\_leapfrog< H, P >::begin\_update\_p | ( | P & | *z*, |
|  |  | H & | *hamiltonian*, |
|  |  | double | *epsilon* |
|  | ) |  |  |

pure virtual

Implemented in stan::mcmc::expl\_leapfrog< H, P >, stan::mcmc::expl\_leapfrog< unit\_e\_metric< M, BaseRNG >, unit\_e\_point >, stan::mcmc::expl\_leapfrog< dense\_e\_metric< M, BaseRNG >, dense\_e\_point >, and stan::mcmc::expl\_leapfrog< diag\_e\_metric< M, BaseRNG >, diag\_e\_point >.

template<typename H, typename P>

|  |  |  |  |
| --- | --- | --- | --- |
| virtual void stan::mcmc::base\_leapfrog< H, P >::end\_update\_p | ( | P & | *z*, |
|  |  | H & | *hamiltonian*, |
|  |  | double | *epsilon* |
|  | ) |  |  |

pure virtual

Implemented in stan::mcmc::expl\_leapfrog< H, P >, stan::mcmc::expl\_leapfrog< unit\_e\_metric< M, BaseRNG >, unit\_e\_point >, stan::mcmc::expl\_leapfrog< dense\_e\_metric< M, BaseRNG >, dense\_e\_point >, and stan::mcmc::expl\_leapfrog< diag\_e\_metric< M, BaseRNG >, diag\_e\_point >.

template<typename H, typename P>

|  |  |  |  |
| --- | --- | --- | --- |
| void stan::mcmc::base\_leapfrog< H, P >::evolve | ( | P & | *z*, |
|  |  | H & | *hamiltonian*, |
|  |  | const double | *epsilon* |
|  | ) |  |  |

inlinevirtual

Implements stan::mcmc::base\_integrator< H, P >.

Definition at line 19 of file base\_leapfrog.hpp.

template<typename H, typename P>

|  |  |  |  |
| --- | --- | --- | --- |
| virtual void stan::mcmc::base\_leapfrog< H, P >::update\_q | ( | P & | *z*, |
|  |  | H & | *hamiltonian*, |
|  |  | double | *epsilon* |
|  | ) |  |  |

pure virtual

Implemented in stan::mcmc::expl\_leapfrog< H, P >, stan::mcmc::expl\_leapfrog< unit\_e\_metric< M, BaseRNG >, unit\_e\_point >, stan::mcmc::expl\_leapfrog< dense\_e\_metric< M, BaseRNG >, dense\_e\_point >, and stan::mcmc::expl\_leapfrog< diag\_e\_metric< M, BaseRNG >, diag\_e\_point >.

template<typename H, typename P>

|  |  |  |  |
| --- | --- | --- | --- |
| void stan::mcmc::base\_leapfrog< H, P >::verbose\_evolve | ( | P & | *z*, |
|  |  | H & | *hamiltonian*, |
|  |  | const double | *epsilon* |
|  | ) |  |  |

inline

Definition at line 30 of file base\_leapfrog.hpp.

---

The documentation for this class was generated from the following file:

- src/stan/mcmc/hmc/integrators/base\_leapfrog.hpp


---

[ Stan Home Page ]

*© 2011–2014,
Stan Development Team.*
